# Application of handheld devices to field research among underserved construction worker populations: a workplace health assessment pilot study

**DOI:** 10.1186/1476-069X-10-27

**Published:** 2011-04-01

**Authors:** Alberto J Caban-Martinez, Tainya C Clarke, Evelyn P Davila, Lora E Fleming, David J Lee

**Affiliations:** 1 Department of Epidemiology & Public Health, Miller School of Medicine, University of Miami, Miami, FL USA

## Abstract

**Background:**

Novel low-cost approaches for conducting rapid health assessments and health promotion interventions among underserved worker groups are needed. Recruitment and participation of construction workers is particularly challenging due to their often transient periods of work at any one construction site, and their limited time during work to participate in such studies. In the present methodology report, we discuss the experience, advantages and disadvantages of using touch screen handheld devices for the collection of field data from a largely underserved worker population.

**Methods:**

In March 2010, a workplace-centered pilot study to examine the feasibility of using a handheld personal device for the rapid health assessment of construction workers in two South Florida Construction sites was undertaken. A 45-item survey instrument, including health-related questions on tobacco exposure, workplace safety practices, musculoskeletal disorders and health symptoms, was programmed onto Apple iPod Touch^® ^devices. Language sensitive (English and Spanish) recruitment scripts, verbal consent forms, and survey questions were all preloaded onto the handheld devices. The experience (time to survey administration and capital cost) of the handheld administration method was recorded and compared to approaches available in the extant literature.

**Results:**

Construction workers were very receptive to the recruitment, interview and assessment processes conducted through the handheld devices. Some workers even welcomed the opportunity to complete the questionnaire themselves using the touch screen handheld device. A list of advantages and disadvantages emerged from this experience that may be useful in the rapid health assessment of underserved populations working in a variety of environmental and occupational health settings.

**Conclusions:**

Handheld devices, which are relatively inexpensive, minimize survey response error, and allow for easy storage of data. These technological research modalities are useful in the collection and assessment of environmental and occupational research data.

## Background

The construction industry is one of the largest industries in the United States, employing over eleven million persons representing 8% of the total labor force [1-3]. Each year, several hundred thousand construction workers become ill or injured as a result of worksite hazards. The estimated rates for injuries, illnesses, and fatalities among construction workers are consistently among the highest of any occupational sector [4]. Construction workers in the U.S. have the highest rate of smoking among all occupations (38% versus 22% for all workers), and also are subject to synergistic occupational exposures such as dust and asbestos, which can further increase lung cancer and chronic lung disease risks [5]. In addition, 43% of U.S. construction workers are overweight, compared to 35% of all workers [6]. Finally, because construction work includes material handling, awkward work postures, and other physical demands, many construction workers also develop work-related musculoskeletal disorders, such as chronic low back pain, shoulder, and other joint conditions [7].

Tailored health promotion interventions and workplace health assessments are powerful tools that increase the relevance and salience of health information by making it workplace and personally relevant [8]. Unfortunately, construction workers have traditionally experienced difficulties participating in worksite-based health promotion programs due to the nature of their work which includes inflexible work schedules and limited breaks[1,2]. In addition, workers are often not situated in one location for long periods of time, but rather may move from one job site to another [7]. Developing novel approaches for conducting construction workplace health assessment and health promotions interventions that close occupational health disparity gaps are urgently needed.

Technical devices, such as handheld computers and text messaging, have been suggested as convenient methods for survey data collection and participant engagement due to the fact that they allow for rapid assessment and information sharing at a relatively low cost [9,10]. For example, Seebregts et al recently documented a windows-based survey software for handheld devices that captures health information in 11 languages and various question formats (e.g. multiple choice, short answer, etc) that resulted in improved data validation, less data cleaning times, and fewer data collection errors in the school setting [9]. In the clinical setting, such as the emergency room, that requires rapid and accurate data capture and interpretation, researchers found improved image (e.g. CT) reading scores between physicians using personal digital assistants as compared to a traditional workstation display[11]. Few environmental and occupational studies have used handheld devices to capture health and exposure information directly at the worksite, particularly among underserved hard-to-reach workers that traditionally encounter inflexible work schedules or financial constraints, such as construction workers.

In the present methodology report, we describe and discuss the experience, advantages and disadvantages of using touch screen handheld devices for the collection of field data from an underserved transient worker population.

## Methods

**Figure 1:**
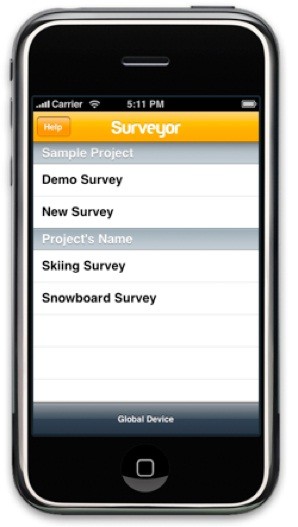
**Interface of Touch Metric's Surveyor software loaded onto Apple's iTouch software**.

A research team comprised of graduate public health students and their faculty mentors undertook a workplace-centered pilot study to examine the feasibility of using a handheld personal device for the rapid health assessment of construction workers. The 45-item survey instrument, which included health-related questions (such as tobacco exposure, workplace safety practices, musculoskeletal disorders and health symptoms), was programmed onto Apple iPod Touch^® ^devices using Touch Metric's Surveyor^® ^software (Figure 1) [12,13]. The vast majority of the selected measures utilized for the workplace health assessment were obtained from well-established surveys such as the National Health Interview Survey (NHIS) [14]. Socio-demographic information on age, gender, race, ethnicity, marital status, educational training, household income, and health insurance status were also collected. Anthropometric measurements collected in the field were also recorded onto the handheld devices when applicable.

**Figure 2:**
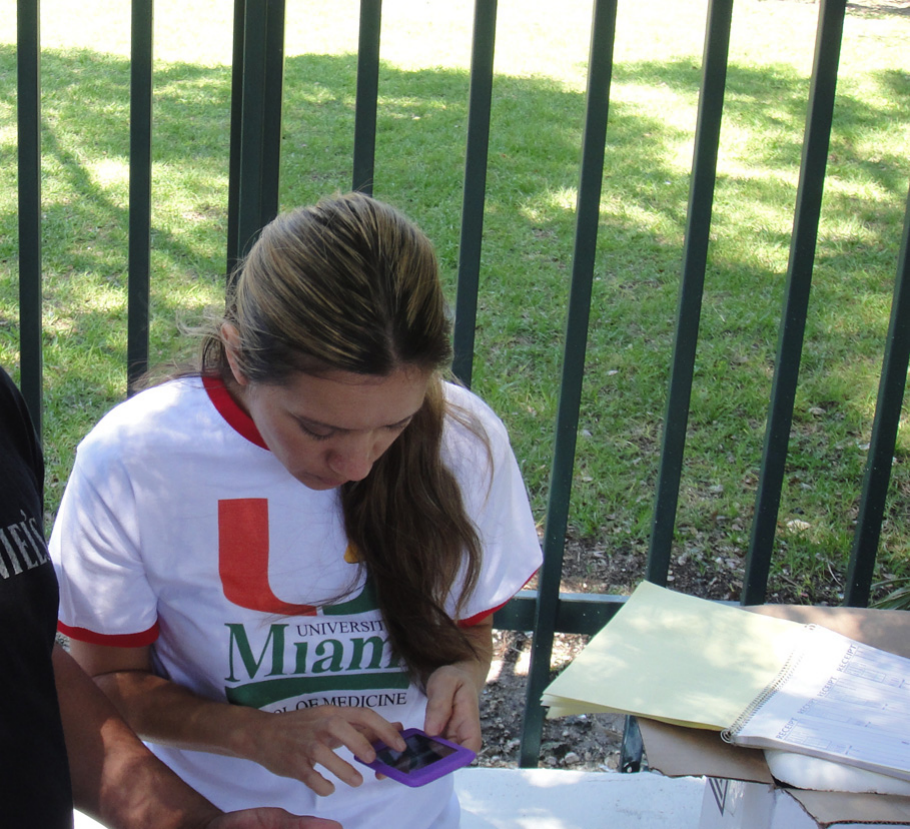
**Student interviewer using touch screen handheld device to record construction worker responses adjacent to lunch truck**.

Language sensitive (English or Spanish) recruitment scripts, verbal consent forms, and survey questions were all preloaded onto the handheld devices. These forms and survey instruments were also made available on-site in paper format in the event a handheld device failed and for researchers who preferred to use paper. In collaboration and conjunction with visitation by a local lunch truck service, the research team recruited participants at two construction sites in South Florida either in the morning during the construction workers 15-minute breakfast break, or at noon during their designated 45-minute lunch break. Bilingual student interviewers approached construction workers congregating around the lunch truck armed only with their handheld devices to provide the construction worker a description of the study, an invitation to participate, verbal consent, and interview administration (Figure 2). The same approach and process was performed by bilingual senior faculty mentors, however they expressed a preference for the paper-based method for data collection. Construction workers completing both the questionnaire and anthropometric measurements were given a $20 incentive along with a drawstring bag loaded with bilingual health educational materials on smoking cessation, construction workplace injury prevention, nutrition, physical activity, and cancer screening.

Across the two construction sites, we approached a total of 57 construction workers that walked to the lunch trunk; 54 enrolled and completed the workplace health assessment pilot study of which 91% self-reported being Hispanic (n = 49). Among the completed assessments, 32 were completed by handheld device, and 22 by traditional paper method. Eleven assessments were administered in English (7 on handhelds and 4 by paper) and 43 in Spanish (25 on handhelds and 18 by paper). For workplace assessments conducted using the handheld devices, it took an average of 9 minutes and 37 seconds to complete the questionnaire; 11 minutes and 8 seconds for the Spanish version, and 9 minutes and 4 seconds for the English version. We did not collect the amount of time needed to administer the paper questionnaires. In terms of data validation, there were no missing or incorrectly entered questionnaire items among all handheld device user entries, while on average 2 items were not intelligibly circled or clearly printed on the paper version of the questionnaire.

## Results

Overall, construction workers were very receptive to the recruitment, interview and assessment processes conducted through the handheld devices. Some workers even welcomed the opportunity to complete the questionnaire themselves using the touch screen handheld device, although ultimately all interviews conducted with handhelds were completed solely by trained study personnel. A list of advantages and disadvantages emerged from this experience that may be useful in the rapid health assessment of underserved populations working in a variety of field settings is displayed in Table 1. Advantages included the initial programming of survey questions into the device was relatively straightforward. The programmer copied ("pasted") the survey questions from a document directly into the survey website, and generated the touchable screen survey directly onto the handheld devices. It took approximately 45-minutes to program the 45-item survey questions into the handheld device. There were non-existent data collection errors (e.g. skipped survey questions) compared to the paper-based option. Finally, data collected from the handheld devices automatically recorded the start and stop times of each interview, the interviewer name, and made data easily downloadable from the handheld device directly onto a pre-formatted excel file with variable column headers (saving data entry time).

**Table 1:** Advantages and disadvantages of using touch screen handheld devices in conducting field workplace health assessments

Advantages	
	No papers (recruitment script, verbal consent form) or pens needed, just the handheld device

	No missing data in the handheld devices, while missing and sometimes unintelligible data on the paper-based option

	Data easily downloaded from device and automatically loaded into Excel spreadsheet

	Clear audit trail of which interviewer administered the protocol and timing of administration

	More usable in inclement weather (using a suitable covering) than paper-based recording system

	Enhanced security features relative to paper data-collection forms (data confidentiality protection)

	Electronic data requires less physical space to store than signed consent or data forms

	Devices are easy to program with web-interface, especially for multiple, simultaneous interview scripts

	No-data entry costs (versus paper-option)

	Construction workers may be interested and willing to complete the interviews themselves using the handheld devices

**Disadvantages**	

	Devices must be powered overnight prior to field data collection

	Keyboard initially cumbersome for some interviewers

	Periodic reviews for residual data remaining on devices and for proper data transmission

	Potential loss of data due to device malfunction

	Lack of acceptance by some researchers

	Initial cost of each handheld device may be moderately high

Some disadvantages should be noted. Senior study interviewers were reluctant to embrace use of the handheld device, citing difficulties with seeing the screens outside in bright light and in pressing the screen question options. In addition, while the devices have very long battery life (~30 hours) between charges, quality checks for battery life were necessary to ensure workability and function in the field. Although the initial start up cost may appear high for each device (~$200), data entry costs were eliminated, making use of handheld devices particularly attractive for researchers conducting multiple, ongoing field assessments. Finally, the relative ease of use combined with the low long-term cost (e.g. monthly subscription fee of $20/month to access web-based Touch Metric's Surveyor^® ^software) of using handheld devices may be one strategy for expanding capacity building within the context of community-based participatory research partnerships [15].

## Discussion

In this pilot study, we found that construction workers were willing to participate in our workplace health assessment study using handheld devices. The speed, flexibility and accuracy of using the handheld devices (as compared to traditional paper-based methods) during the construction workers breakfast and lunch breaks at the lunch truck may reduce commonly encountered barriers to real-time workplace assessments. Future research in the application of these devices among time-constrained worker groups could improve engagement and retention in workplace health promotion programs for occupational groups that do not have a formal workplace setting. Furthermore, the interest and acceptance of these devices expressed by the workers in this study might lead to the use of self-administered questionnaires on handheld personal devices in future studies.

## Conclusion

From our field experience, we concluded that the use of a handheld personal device designed for time-pressed and hard to reach workers is a cost-effective and flexible modality for data collection that can assist with the rapid engagement of a traditionally difficult-to-reach vulnerable worker population. This methodology report adds to the extant literature on the advantages and disadvantages of using handheld devices in among hard to reach worker populations. Public health practitioners are encouraged to embrace informatics tools that enhance public health practice.

## List of abbreviations

US: United States; NHIS: National Health Interview Survey

## Competing interests

The authors declare that they have no competing interests.

## Authors' contributions

AJCM was responsible for conception, acquisition of funding, and general supervision of the research group. AJCM, TCC, EPD, LEF, and DJL made substantial contributions to data collection and study design. AJCM, TCC, EPD, LEF, and DJL contributed to analysis and interpretation of data; AJCM, TCC, EPD, LEF, and DJL were involved in drafting the manuscript or revising it critically for intellectual content. All authors read and approved the final manuscript.

## Photography Consent

The study authors have obtained and keep on file photographic consent for Figure 2 photos (including photo subjects' consent) that will appear in this paper.
